# Survival and axillary recurrence following sentinel node-positive breast cancer without completion axillary lymph node dissection: the randomized controlled SENOMAC trial

**DOI:** 10.1186/s12885-017-3361-y

**Published:** 2017-05-26

**Authors:** Jana de Boniface, Jan Frisell, Yvette Andersson, Leif Bergkvist, Johan Ahlgren, Lisa Rydén, Roger Olofsson Bagge, Malin Sund, Hemming Johansson, Dan Lundstedt, on behalf of the SENOMAC Trialists’ Group, Peer Christiansen, Tove Filtenborg Tvedskov, Birgitte Offersen, Toralf Reimer, Thorsten Kühn, Michalis Kontos, Oreste Gentilini

**Affiliations:** 1 Department of Surgery, Capio St Göran’s Hospital, Stockholm, Sweden; 2 Department of Molecular Medicine and Surgery, Karolinska Institutet, Stockholm, Sweden; 3 Department of Breast and Endocrine Surgery, Karolinska University Hospital, Stockholm, Sweden; 4 Department of Surgery, Västmanland County Hospital, Västerås, Sweden; 5 Center for Clinical Research, Uppsala University, Västmanland County Hospital, Västerås, Sweden; 6 Department of Oncology, University of Örebro, Örebro, Sweden; 7 Department of Surgery, Institution of Clinical Science, Lund University, Lund, Sweden; 8 Department of Surgery, Skåne University Hospital, Lund, Sweden; 9 Department of Surgery, Institute of Clinical Sciences, Sahlgrenska Academy at the University of Gothenburg, Sahlgrenska University Hospital, Gothenburg, Sweden; 10 Surgery Center, Norrland University Hospital, Umeå, Sweden; 11 Department of Surgical and Perioperative Science, Umeå University, Umeå, Sweden; 12 Department of Oncology-Pathology, Clinical Trials Office, Karolinska Institutet, Stockholm, Sweden; 13 Department of Oncology, Sahlgrenska University Hospital, Gothenburg, Sweden

**Keywords:** Breast cancer, Sentinel lymph node biopsy, Axillary lymph node dissection, Survival, Macrometastasis

## Abstract

**Background:**

The role of axillary lymph node dissection (ALND) has increasingly been called into question among patients with positive sentinel lymph nodes. Two recent trials have failed to show a survival difference in sentinel node-positive breast cancer patients who were randomized either to undergo completion ALND or not. Neither of the trials, however, included breast cancer patients undergoing mastectomy or those with tumors larger than 5 cm, and power was debatable to show a small survival difference.

**Methods:**

The prospective randomized SENOMAC trial includes clinically node-negative breast cancer patients with up to two macrometastases in their sentinel lymph node biopsy. Patients with T1-T3 tumors are eligible as well as patients prior to systemic neoadjuvant therapy. Both breast-conserving surgery and mastectomy, with or without breast reconstruction, are eligible interventions. Patients are randomized 1:1 to either undergo completion ALND or not by a web-based randomization tool. This trial is designed as a non-inferiority study with breast cancer-specific survival at 5 years as the primary endpoint. Target accrual is 3500 patients to achieve 80% power in being able to detect a potential 2.5% deterioration of the breast cancer-specific 5-year survival rate. Follow-up is by annual clinical examination and mammography during 5 years, and additional controls after 10 and 15 years. Secondary endpoints such as arm morbidity and health-related quality of life are measured by questionnaires at 1, 3 and 5 years.

**Discussion:**

Several large subgroups of breast cancer patients, such as patients undergoing mastectomy or those with larger tumors, have not been included in key trials; however, the use of ALND is being questioned even in these groups without the support of high-quality evidence. Therefore, the SENOMAC Trial will investigate the need of completion ALND in case of limited spread to the sentinel lymph nodes not only in patients undergoing any breast surgery, but also in neoadjuvantly treated patients and patients with larger tumors.

**Trial registration:**

NCT 02240472, retrospective registration date September 14, 2015 after trial initiation on January 31, 2015.

## Background

Lymph node metastasis is one of the factors of greatest prognostic importance in breast cancer [1–3]. Lymph node metastases are classified as isolated tumor cells (≤ 0.2 mm and/or <200 cells), micrometastasis (> 0.2 but ≤2 mm and/or >200 cells) and macrometastasis (> 2 mm) [4]. Sentinel node (SN) biopsy has proven to be a reliable method [5], and several follow-up studies have shown that it is safe to refrain from completion axillary lymph node dissection (ALND) in sentinel node-negative breast cancer [6–10]. The greatest advantage of the SN biopsy approach is the significant decrease in the frequency and severity of arm problems since fewer lymph nodes are removed from the axilla [11–14].

In SN-positive patients, no additional metastases are found in the remaining lymph nodes removed on ALND in about 50–65% of patients [15]. After the publication of the ACOSOG Z0011 trial in 2011 [16], refraining from completion ALND in SN-positive cases has been embraced widely especially in the US [17, 18]. This trial randomized SN-positive patients to either undergo ALND or to refrain from completion axillary surgery. After a median follow-up period of over 6 years, no difference in the rate of axillary recurrence was found, and survival was even slightly better among patients who only underwent SN biopsy (disease-free survival 83.9%, compared with 82.2% for patients who underwent ALND), although the difference was not statistically significant. The study has received some criticism [19, 20]. ACOSOG Z0011 only included patients with tumors up to 5 cm in size who underwent breast-conserving surgery, receiving whole-breast postoperative radiotherapy.

Another study (IBCSG 23–01), in which SN-positive patients were randomized to either undergo completion ALND or not, was published in 2013 [21]. This study included only patients with SN micrometastases, but also showed slightly better disease-free survival in the group operated with SN biopsy alone (87.8% compared with 84.4% for those who underwent ALND), though the difference was not statistically significant here either. Neither the ACOSOG Z0011 study nor the IBCSG 23–01 study succeeded in enrolling the planned number of patients and the studies do not have sufficiently high power to detect small differences.

There are a few studies suggesting that ALND may still have some therapeutic benefit: The rate of axillary recurrence among SN-positive patients who did not undergo ALND was a striking 2.0% after just 30 months, despite otherwise favorable prognostic factors (compared with 0.4% among those who underwent completion ALND) in a report by Park et al. [22]. In the Dutch MIRROR study [23] the rate of axillary recurrence was more than twice as high among patients with SN micrometastases who did not undergo ALND compared with SN-negative patients.

In Sweden, most patients with SN macrometastases receive adjuvant radiotherapy to the axillary region. A large European study (AMAROS) randomizing over 1400 SN-positive patients, of whom 861 with SN-macrometastases, to either undergo completion ALND or to have axillary radiotherapy showed no difference in disease-free or overall survival [24]. Subsequently, several countries now approve axillary radiotherapy in lieu of axillary lymph node dissection.

None of the described trials included a sufficient amount of patients treated by mastectomy to draw any conclusions on the need of ALND. It is also unclear whether the tumor size should be limited to 5 cm at most or whether larger, although not locally advanced tumors may be included along the same line of thought. Finally, as the rates of breast cancer treated by neoadjuvant systemic therapy (NAST) are rising internationally, the question how to surgically treat the axilla post NAST in the event of a positive pre-NAST SN biopsy needs to be answered. The SENOMAC trial attempts to answer these highly important questions in an international collaborative effort.

## Methods

This prospective multicenter non-inferiority trial randomizes breast cancer patients with macrometastasis in at most two sentinel nodes to either undergo completion ALND (arm A) or not to have any further axillary surgery (arm B), and is conducted according to Good Clinical Practice (GCP) guidelines. For inclusion and exclusion criteria, see Table 1.

**Table 1:** Inclusion and exclusion criteria according to the SENOMAC study protocol

Inclusion criteria	Primary invasive breast cancer T1-T3^a^
	Preoperative ultrasound of the axilla performed
	Macrometastasis in not more than two lymph nodes at sentinel node biopsy
	Written informed consent
	Age 18 years or older
Exclusion criteria	Palpable regional lymph node metastasis prior to surgery
	Regional or distant metastases outside of the ipsilateral axilla
	Pregnancy
	Bilateral invasive breast cancer, if one side meets any exclusion criteria
	Medical contraindication for radiotherapy or systemic treatment
	Inability to absorb or understand the meaning of the study information; for example, through disability, inadequate language skills or dementia
	Prior history of invasive breast cancer

^a^According to the TNM classification system

### Aims and endpoints

The main aim of this study is to evaluate whether it is safe to refrain from completion ALND in individuals with breast cancer and SN macrometastasis. Primary endpoint is breast cancer-specific survival at 5 years. Secondary endpoints are locoregional recurrence, disease-free survival and overall survival, but also arm morbidity, health economic outcome and health-related quality of life.

### Preoperative assessment

**Fig. 1:**
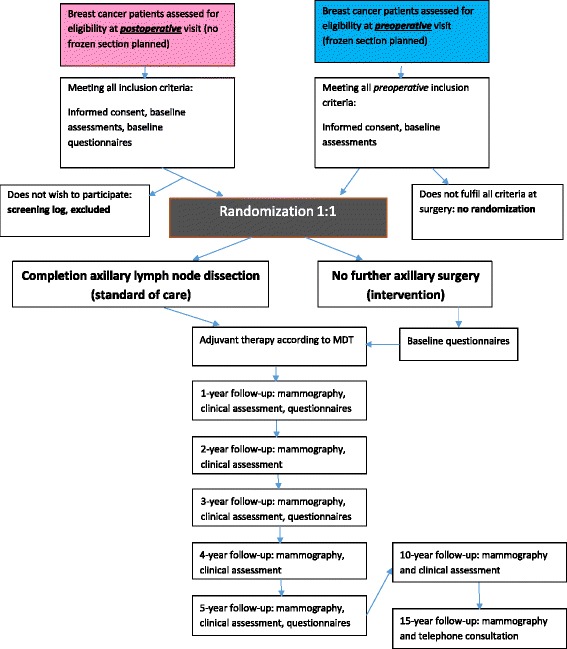
Flow chart of inclusion pathways in the SENOMAC Trial depending on the use of frozen section at sentinel node biopsy

Preoperative assessment is carried out in accordance with local practice with triple diagnostics, namely clinical assessment, imaging evaluation and cytological or histopathological confirmation of the diagnosis. Ultrasound of the axillary region is required and suspicious nodes must be biopsied. Patients with up to two non-palpable preoperatively diagnosed axillary metastases may nevertheless undergo SN biopsy and be included. All types of breast surgery are eligible in this trial. Frozen section may be performed or omitted in the study which warrants different logistic considerations, see Fig. 1.

### Neoadjuvant systemic therapy

Patients planned for neoadjuvant systemic therapy (NAST) may be included in this trial in case all inclusion criteria are met. Thus, patients without palpable lymph node metastases may undergo SN biopsy prior to start of their neoadjuvant treatment. Eligible patients may be randomized and included in this trial. Randomization is recommended to be performed before start of neoadjuvant therapy but must at the very latest take place before the first clinical or radiological response evaluation. In case of tumor progression during NAST and/or the appearance of palpable lymph node metastases, participation in the trial is discontinued and the reason for study termination recorded in the electronic Case Report Form (eCRF). The decision to discontinue participation in the trial should always be discussed at a multidisciplinary team conference.

### Randomization

Web-based randomization may occur either after the receipt of the frozen section results during surgery or after receipt of the final histopathological results postoperatively. Patients randomized to arm A will undergo completion ALND of levels I and II, which may be performed either at the same session (when randomization is based on frozen section results) or in a second session. Patients randomized to arm B will not undergo further axillary surgery.

Randomization is based on permutated block technique and performed 1:1; treatment arms are stratified per country. In the event that the final histopathology results show that a randomized patient does not meet all criteria (e.g., additional metastasis identified during SN sectioning), the patient must be excluded. Patients who fulfil all inclusion criteria and receive information about the trial but are not randomized are to be registered in screening logs on site.

### Questionnaires

Questionnaires regarding arm morbidity, health-related quality of life and health economy will be provided at baseline as well as after 1, 3 and 5 years. The instruments used are the Lymphedema Functioning, Disability and Health Questionnaire (Lymph-ICF) [25], the EQ-5D-5 L utility scores [26], and EORTC’s well-validated QLQ-30 [27, 28] and BR-23 [29] questionnaires. Apart from the traditional paper version, online versions of all instruments will be made available. Answers are coded with an individual study code and collected centrally at the Study Center in Stockholm.

### Adjuvant therapy

Adjuvant systemic therapy should be given in accordance with national clinical guidelines of each participating country. After breast-conserving surgery, the remaining ipsilateral breast parenchyma must be irradiated. Boost to the tumor bed should be applied according to each country’s national guidelines. Post-mastectomy radiotherapy (PMRT) and radiotherapy to the regional lymph node basins are based on each country’s national guidelines. It is, however, mandatory, that for those participating in this trial, radiotherapy should not be extended or changed based on which arm the patient is randomized to, ie, sentinel node biopsy only should be regarded as a substitute for axillary clearance.

In Sweden, radiotherapy to regional lymph node basins follows the recommendations of the Swedish National Guidelines. The regional lymph node target (CTV) is composed of axilla level 2 and 3, interpectoral lymph nodes and supraclavicular fossa (i.e. axilla level 4) which means that level 1 is omitted from the regional lymph node CTV. For detailed volume description, please see the target definition at ESTRO consensus guideline on target volume delineation for elective radiation therapy of early stage breast cancer, version 1.1. [30].

The exact regional lymph node target is to be reported in the eCRF prospectively throughout the trial. Irradiation of internal mammary nodes (IMN) should be handled according to national guidelines of each country and treatment of IMN must be recorded in the eCRF.

Fractionation schedule is chosen according to local practice, i.e. 2 Gy/f × 25 over 33–35 days to the breast and regional lymph nodes. A slightly lower total dose to the nodes (~46 Gy) is accepted. Hypofractionated radiotherapy can be chosen, i.e. 2.67 Gy/f × 15–16 over 19–22 days. Dose and fractionation is to be reported prospectively.

### Data management

All data are registered using an electronic Case Report Form (eCRF). Monitoring is performed according to Good Clinical Practice (GCP) guidelines. In the eCRF, data on age, completed surgery, tumor and lymph node characteristics, as well as neoadjuvant and adjuvant therapy are collected, as well as status at annual follow-up. Data are managed by the Clinical Trial Unit at Karolinska University Hospital, Stockholm, Sweden.

### Monitoring and follow-up

Patients will be followed by annual clinical examination and mammography for 5 years.

Each follow-up visit must take place within +/− 2 months from the randomization date, and data are to be completed in the eCRF within 1 month from the follow-up visit. Additional diagnostic measures, e.g. axillary ultrasound, biopsies or other investigations, are carried out according to clinical findings. In case of suspected axillary recurrence, a CT of the thoracic region is requested in order to define the level of recurrence in the axilla and exclude further metastatic spread.

### Sample size

The goal of the study is to establish that the intervention (no further axillary surgery) is statistically non-inferior to standard of care (completion ALND) for the primary endpoint breast cancer-specific survival (BCSS) at 5 years.

Clinical non-inferiority is in this study defined as a 5-year BCSS not worsened by more than 2.5% when refraining from ALND. To show that (i.e. a 5-year BCSS of 89.5% in the intervention group compared to 92% in the standard of care group - using a one-sided α of 10% and with a power of 80%) a total of 225 breast cancer deaths need to be observed in the study. This corresponds to show that the upper one-sided 90% confidence interval for the hazard ratio (HR: Intervention/Standard of care) falls below 1.33. Power calculations are based on Swedish data which may differ from survival outcomes in other countries. Therefore, stratification according to country of primary treatment is performed.

It is anticipated that the study will be able to recruit up to 700 patients per year during a 5-year period giving a total sample size of 3500 patients. With allowance for an extra year of follow-up the necessary number of events (225) is expected to be reached. The total study time will be approximately 7 years.

### Data monitoring committee

An independent data monitoring committee will review the data and carry out one closed interim analysis 3 years after the date on which the first study patient was randomized, or when 2000 patients have been included in the study, whichever comes first. The purpose of this interim analysis is to assess the recruitment to the study, the rate of overall breast-cancer related events and to make sure that patients in the intervention group do not appear to fare significantly worse than patients in the standard of care group. The committee may recommend terminating the study if a significant benefit in favour of standard of care for breast-cancer deaths is shown, such that the HR for intervention versus standard of care significantly (*p* = 0.001) exceeds 1, or if the recruitment is so low that that the necessary number of events is unlikely to be reached. If the committee determines that it is safe to proceed with the study, the results of the analysis will remain unknown to everyone except the committee members.

### Statistical methods

For the primary endpoint breast cancer-specific survival, time will be calculated from the date of randomization to the date of breast cancer death (BCD). A breast cancer death will be defined as a death with information of a preceding or concurrent regional or distant recurrence. Isolated ipsilateral in-breast recurrences will thus not count towards BCD. Disease-free survival time is calculated from the date of randomization to the date of loco-regional recurrence, date of distant recurrence, date of second malignancy or date of death, whichever comes first. For event-free patients time will be calculated from the date of randomization to the date of last visit.

Event-specific cumulative incidence rates - taking competing risks into account - will be estimated using non-parametric methods. Differences in time to failure will be tested using the log-rank test. The effect of the intervention on time to failure will be estimated using proportional hazards regression. Both unadjusted analyses and analyses adjusting for potential confounding factors will be performed. Longitudinal health-related quality of life data will be analysed using generalized linear models. Test for interactions between treatment and time – indicating a differential effect of treatment over time – will also be performed. Both intent-to-treat analyses and treatment received analyses will be performed for the primary outcome.

All analyses will be performed using StataCorp 2015 (College Station, TX: StataCorp LP).

## Discussion

Despite a general decline in the use of ALND after the first publication of the results from the ASOSOG Z0011 trial in 2011 [16], there is still considerable variation in surgical management of the axilla across European centers [31]. Even after the recent publication of long-term results from the same study, showing essentially no difference in recurrence rates between patients undergoing or omitting completion ALND [32], the base of evidence remains small. A review by Schmidt-Hansen et al. [33] identified only three prospective randomized trials comparing SN-biopsy with or without completion ALND in patients with SN metastases, reporting on a total of 2020 patients. This said, two of these three trials did exclusively include cases of SN-micrometastasis (AATRM 048/13/2000 and IBCSG-23-01) and the third trial (ACOSOG Z0011) included only 430 patients with SN-macrometastases while the remaining 301 patients had SN-micrometastases only; the size of SN-metastasis was not reported on the 125 patients left in the intent-to-treat sample (*N* = 856). Thus, evidence on the significance of completion ALND in patients with SN-macrometastasis is limited to a small sample of individuals with T1-T2 tumors treated by breast-conserving surgery. Despite this, the use of ALND in SN-positive disease seems to decrease even in patients treated by mastectomy [34]; in other instances, ALND may be replaced by regional radiotherapy after the results of the AMAROS trial [24]. This clearly leaves a need for further prospective trials, including patients treated by mastectomy.

In the setting of neoadjuvant systemic therapy (NAST), it has been argued that SN biopsy pre NAST has the disadvantage to necessitate a second axillary intervention (ALND) in case of SN-macrometastases in cN0 patients [35]. The false negative rate in repeat SN biopsy after NAST is high [36] but is acceptable if performed primarily after NAST in case at least three SN can be identified. While the latter has become routine in some countries, SN biopsy is still performed prior to NAST in Sweden and other countries in case of clinical node negativity. The SENOMAC trial gives the opportunity to benefit from the up-front staging information the SN biopsy can offer in cN0 patients while investigating whether a completion ALND is necessary in those with up to two SN macrometastases. Thus, this trial may conclude whether or not ALND is at all indicated in patients with pre NAST SN macrometastases, given that no tumor progression is observed during NAST.

In summary, the SENOMAC trial aims to answer the clinically pending questions concerning the indications for ALND in T1-T3 tumors in cN0 patients. Importantly, it not only includes both breast conservation and mastectomy but also patients selected for neoadjuvant systemic therapy.

## Abbreviations

ACOSOG: American College of Surgeons Oncology Group
ALND: Axillary lymph node dissection
BCD: Breast cancer death
BCSS: Breast cancer-specific survival
CT: Computed tomography
CTV: Clinical target volume
eCRF: Electronic Case Report Form
EORTC: European Organisation for Research and Treatment of Cancer
ESTRO: European SocieTy for Radiotherapy and Oncology
GCP: Good Clinical Practice
HR: Hazard ratio
IBCSG: International Breast Cancer Study Group
IMN: Internal mammary nodes
NAST: Neoadjuvant systemic therapy
PMRT: Post-mastectomy radiotherapy
SN: Sentinel Node
